# PET evaluation of light-induced modulation of microglial activation and GLP-1R expression in depressive rats

**DOI:** 10.1038/s41398-020-01155-z

**Published:** 2021-01-06

**Authors:** Yu Liu, Lizhen Wang, Donghui Pan, Mingzhu Li, Yaoqi Li, Yan Wang, Yuping Xu, Xinyu Wang, Junjie Yan, Qiong Wu, Lin Lu, Kai Yuan, Min Yang

**Affiliations:** 1 NHC Key Laboratory of Nuclear Medicine, Jiangsu Key Laboratory of Molecular Nuclear Medicine, Jiangsu Institute of Nuclear Medicine, Wuxi, 214063 Jiangsu China; 2 Peking-Tsinghua Center for Life Sciences, Peking University, 100871 Beijing, China; 3 Peking University Sixth Hospital, Peking University Institute of Mental Health, NHC Key Laboratory of Mental Health (Peking University), National Clinical Research Center for Mental Disorders (Peking University Sixth Hospital), Peking University, 100191 Beijing, China

**Keywords:** Biomarkers, Depression, Molecular neuroscience

## Abstract

Light therapy has been accepted as a promising therapeutic choice for depression. Positron emission tomography (PET) combined with specific radiotracers has great benefits for revealing pathogenesis and developing therapeutics. This study aimed to investigate the influences of light therapy on microglial activation and glucagon-like peptide-1 receptor (GLP-1R) expression in the brain of depressive rats using [^18^F]DPA-714 and [^18^F]exendin-4 PET. The results showed that chronic unpredictable mild stress (CUMS)-induced depressive rats had poorer performance in behavioral tests compared to normal rats (*p* < 0.05) and the depressive-like behavior could be ameliorated by light therapy. Besides, depressive rats had significantly higher [^18^F]DPA-714 uptake and lower [^18^F]FDG uptake compare to normal rats in 11 and 9 regions of interest (ROIs) of the brain, respectively (*p* < 0.05). After 5 weeks of light therapy, higher [^18^F]FDG and [^18^F]exendin-4 uptake was observed in most ROIs of light therapy-treated depressive rats compared to untreated depressive rats (*p* < 0.05) and no significant differences existed in [^18^F]DPA-714 uptake between the two groups. This study demonstrated that light therapy can ameliorate depressive-like behavior, improve glucose metabolism, and halt the decline of brain GLP-1R expression of depressive rats, but have no effects on microglial activation caused by CUMS. Besides, this study validated that [^18^F]DPA-714 and [^18^F]exendin-4 PET have the potential for noninvasive evaluation of microglial activation and GLP-1R expression in the brain of depression.

## Introduction

In the past few years, light therapy has been accepted as an optional therapeutic choice for some neuropsychiatric disorders such as major depressive disorder, circadian phase sleep disorder, Parkinson’s disease, and Alzheimer’s disease^1–5^. However, the mechanisms of light therapy for depression remain unclear and there is a lack of efficacy evaluation methods for this treatment. Positron emission tomography (PET) is a well-recognized imaging method for noninvasive diagnosis and efficacy evaluation of neuropsychiatric disorders, besides, PET combined with specific radiotracers has great benefits for revealing pathogenesis and developing therapeutics. For example, recent studies demonstrated the reduced cerebral MAO-A levels and serotonin transporter binding potential in patients with depression after a few weeks of bright light therapy using [^11^C]harmine and [^11^C]DASB PET imaging^6,7^. However, the complex pathology of depression makes it still possesses many other important targets worth exploring.

The inflammatory hypothesis in depression has received lots of attention in recent years. Numerous studies illustrated that depression may be associated with microglial activation and neuroinflammation^8,9^. However, whether light therapy has effects on neuroinflammation in depression remains controversial^10,11^. The 18 kDa translocator protein (TSPO) found on the outer mitochondrial membrane has been considered to be a reliable biomarker of neuroinflammation because its expression is low in healthy brain under physiological conditions, but significantly elevated in activated microglia under inflammatory^12^. Over the past three decades, dozens of radioligands specifically target TSPO have been synthesized and some of them were tested in preclinical and clinical studies of depression^13–17^. [^18^F]DPA-714 is a second-generation TSPO radiotracer that has higher signal-to-noise ratio and lower nonspecific uptake in the brain compared to the currently widely used [^11^C]PK11195^18^. Besides, ^18^F has a longer half-life (109.8 min) compared to ^11^C (20.4 min) which is more suitable for clinical application. Due to its promising properties, [^18^F]DPA-714 has been investigated in many pathological conditions including stroke^19^, epilepsy^20^, and Alzheimer’s disease^21^. However, few studies have explored its potential in depression. Our previous study suggested that rats with depression might have elevated [^18^F]DPA-714 uptake in the hippocampus, but the more comprehensive uptake profile and imaging performance of [^18^F]DPA-714 PET in the whole brain of depression need to be further investigated^22^.

Accumulating data suggested that altered gut microbiota could have influences in psychiatric disorders^23^. The glucagon-like peptide-1 (GLP-1), a 30-amino acid peptide hormone which is mainly produced by intestine L cells, has been considered to be a mediator in the bidirectional microbiota–gut–brain axis^24^. Several studies have demonstrated that GLP-1 receptor (GLP-1R) agonists share some key enzymes and signaling pathways with antipsychotic drugs^25–27^ and a recent meta-analysis described the antidepressant effects of GLP-1R agonists^28^. Besides, studies also showed that GLP-1R agonists as well as some probiotics could improve depressive behaviors by upregulating GLP-1R expression in the brain^29,30^. However, no studies have explored whether light therapy can mediate cerebral GLP-1R expression. Besides, a handful of studies have evaluated the distribution and expression of GLP-1R in the central nervous system using immunohistochemistry^31^. However, no reliable imaging methods have been developed for the noninvasive visualization and quantification of brain GLP-1R expression. In our previous study, we firstly evaluated the feasibility of GLP-1R PET in the brain using [^18^F]AlF-NOTA-MAL-Cys^39^-exendin-4 ([^18^F]exendin-4) and demonstrated the influences of age on GLP-1R expression^32^. However, a comprehensive evaluation in the performance of GLP-1R PET should be conducted and more adequate imaging parameters should be provided to prove that GLP-1R PET can be used for brain imaging in specific pathology and provide a reference for further improvement of this imaging method.

In summary, this study aimed to investigate whether chronic unpredictable mild stress (CUMS)-induced depression rat model has altered microglial activation and brain GLP-1R expression using PET imaging and describe the imaging patterns of specific radiotracers [^18^F]DPA-714 and [^18^F]exendin-4 in depressive rats. Furthermore, we would like to explore whether microglial activation and brain GLP-1R expression could be mediated by light therapy in depressive rats using these PET imaging methods. Here, we would additionally use [^18^F]FDG PET as a reference, which is used for the evaluation of glucose metabolism and is the most widely accepted and used PET tracer in the clinic^33^.

## Materials and methods

### Animals

Sprague-Dawley rats (male, 5–6 weeks, 250–300 g) used in this study were purchased from Changzhou Cavens Laboratory Animal Co., Ltd. (Changzhou, China) and housed in the Laboratory Animal Center of Jiangsu Institute of Nuclear Medicine under a temperature-controlled (23 ± 2 °C) and humidity-controlled (~50%) condition. Rats were free to access food and water and the room was in a 12 h light/dark cycle (7:00 a.m. to 7:00 p.m.). Sample size was determined based on previous published data as sufficient to obtain statistical significance. All animal experiments involved in this study were approved by the Laboratory Animal Management and Ethics Committee of Jiangsu Institute of Nuclear Medicine.

### CUMS protocol

Rats were randomly selected for modeling. The CUMS protocol was performed to induce a rodent model of depression^34^. Briefly, rats were housed individually and exposed to one of the following stressors: food deprivation for 24 h; water deprivation for 24 h; clamp the tail for 5 min; overnight illumination; swimming in cold water (4 °C) for 5 min; ultrasound stimulation (110 dB) for 1 h; tilt the cage at 45° for 12 h; hot temperature (45 °C) for 5 min; cage shaking (1 time/s) for 15 min; electrical stimulation (1 mA, 30 V, 30 s stimulation with 1 min interval) on the sole for 5 min; wet environment for 12 h. The stressor given each day was random and the whole protocol lasted for 4 weeks.

### Behavioral tests

The behavioral tests were performed according to previous studies^35^. For sucrose preference test (SPT), rats were trained to adapt and learn to drink 1% sucrose solution. Then, all rats were given two bottles at the same time: one bottle of 1% sucrose water and one bottle of tap water. The positions of the two bottles were changed every 12 h. The sucrose water and tap water consumed every 24 h were recorded and sucrose preference was calculated as sucrose preference (%) = sucrose water consumption/(sucrose water + tap water consumption). For forced swimming test (FST), rats were placed in a transparent cylindrical bucket of water (depth of 35–40 cm, diameter of 30 cm, and temperature of 22–24 °C). The immobile time of each rat in the water was blindly recorded by two independent observers. The total duration is 6 min and the total immobility was calculated using the data of the last 5 min. For open field test (OFT), rats were placed in a customized box (50 cm × 50 cm × 45 cm) in a room with dim light. The movement of rats within 5 min was recorded with an infrared camera and the total movement distance was measured. Rats induced by CUMS and verified by behavioral tests to have depressive-like behaviors were included as depression rats (CUMS group), otherwise they would be excluded.

### Radiochemistry

The radiolabeling precursor DPA-714 was generously donated by Professor Mengchao Cui of Beijing Normal University. Cys^39^-exendin-4 was purchased from Apeptide Co., Ltd. (Shanghai, China). NOTA-MAL was purchased from CheMatech (Dijon, France). The [^18^F]FDG was provided by Wuxi Fourth People’s Hospital and had satisfied the clinical standard. The ^18^F^–^ was produced on the cyclotron (Sumitomo Heavy Industries, Japan) of Jiangsu Institute of Nuclear Medicine.

The radiosynthesis of [^18^F]DPA-714 and [^18^F]exendin-4 was conducted as described previously with some modifications^36,37^. Briefly, for [^18^F]DPA-714, the ^18^F^−^ produced by the cyclotron was captured on a QMA cartridge (Waters, USA) and eluted into a 5 mL reaction vessel by 1.5 mL K_2.2.2_/K_2_CO_3_. The mixed solution was dried three times under nitrogen flow at 100 °C wherein 1.5 ml of extra-dry acetonitrile (Acros Organics, Belgium) was added each time. Then, 3 mg of DPA-714 stored under vacuum was dissolved in 0.6 ml of extra-dry acetonitrile and added to the completely dried ^18^F^−^ and reacted at 97 °C for 10 min. The reacted solution was cooled to room temperature and injected into a semi-preparative HPLC (Waters, USA). The mobile phase was deionized water with 0.1% triethylamine (SCR, China) and acetonitrile (SCR, China) with 0.1% triethylamine (50/50, v/v) and the flow rate was 5 mL/min. The collected [^18^F]DPA-714 fraction was injected into a C18 cartridge (Waters, USA) and then eluted using 0.6 ml of ethanol (SCR, China) to get the final product. The entire synthesis process could be completed within 1 h while the radiochemical purity is ≥99% and the yield is ~30%. For [^18^F]exendin-4, the NOTA-MAL-Cys^39^-exendin-4 synthesized according to the previous study^37^ was dissolved in deionized water, and then the AlCl_3_ solution, acetonitrile, and glacial acetic acid (Sigma-Aldrich, USA) was added in order, finally the ^18^F^-^ solution was added, then the reaction was conducted at 100 °C for 10 min. The product was captured onto a C18 cartridge using sterile water and finally rinsed off with HCl-containing ethanol. The entire process could be finished within 30 min with a radiochemical purity >95% and yield around 25%.

### PET scans

PET scans were conducted on an Inveon microPET scanner (Siemens Medical Solutions, Germany) and the protocol was based on previous studies^19,20^. Rats were anesthetized with the isoflurane/O_2_ mixture (induction, 4%; maintenance, 2–2.5%). For [^18^F]FDG, rats were fasted one night before PET scan. The 10 min static images were acquired at 60 min after intravenous injection of [^18^F]DPA-714 or [^18^F]FDG, and 30 min after injection of [^18^F]exendin-4, respectively. To obtain dynamic PET images of [^18^F]DPA-714 and [^18^F]exendin-4, four depressive rats were randomly selected and the image acquisitions were continued from the start of the injection to 60 min after injection. For the blocking experiments, blocking dose (5 mg/kg body weight) of unlabeled DPA-714 and Cys^39^-exendin-4 were intravenously injected 10 min before the dynamic acquisitions. The dynamic data were sorted into 31 frames (30 s × 10, 60 s × 5, 120 s × 10, 300 s × 6). Images were normalized, corrected, and reconstructed using Fourier rebinning (FORE) and 3-dimensional ordered subsets expectation maximum (OSEM 3D) algorithm (128 × 128 matrix size, 2 OSEM iterations, and no scatter correction).

### Imaging data analysis

PMOD software was used for all image processing. PET images were co-registered to the anatomical data of the MRI rat brain template provided by PMOD. Thirty-three regions of interest (ROIs) in the brain were drawn automatically. The standard uptake value (SUV) was calculated for static images. For dynamic images, SUV was calculated for each ROI of all frames, and the non-displaceable binding potential (BP_ND_) of each ROI was generated with SRTM2 using the normalized data from blocking group (BP_ND_-BLO) and cerebellum gray matter (BP_ND_-CBL) as the pseudo-reference region, respectively. Parametric images were also derived using BP_ND_-BLO and BP_ND_-CBL.

### Western blot

The rats’ hippocampi tissue was homogenized in RIPA lysis buffer (Beyotime, China). Protein samples were run on 12% Bis-Tris gels (Invitrogen, USA) and transferred to PVDF membranes (Beyotime, China). Primary antibodies against anti-Iba-1 (1:200, Abcam, USA), anti-IL-1β (1:1000, ABclonol, China), anti-IL-6 (1:1000, ABclonol, China), anti-TNF-α (1:200, ABclonol, China), anti-GLP-1R (1: 1000, GeneTex, USA), and anti-β-actin (1:1000, Beyotime, China) were incubated overnight at 4 °C. Secondary antibodies (1:1000–1:10,000, Beyotime, China) were incubated for 2 h at room temperature.

### Immunofluorescence

The brain tissue of rats was taken out after perfusion and made into 12 μm frozen sections. Sections were permeabilized and blocked in PBS containing Triton X-100 and serum. Sections then were incubated with primary antibodies overnight at 4 °C. The primary antibodies including anti-Iba-1 (1:100, Abcam, USA), anti-IL-1β (1:100, ABclonol, China), anti-IL-6 (1:100, ABclonol, China), and anti-GLP-1R (1:100, GeneTex, USA). Secondary antibodies (1:1000, ThermoFisher, USA) were used for sections for 1 h in the dark. Sections were finally observed with an Olympus fluorescent inverted microscope.

### Light therapy protocol

Rats were randomly selected for light therapy (LT group). Briefly, rats were placed in transparent cages in customized opaque boxes. Blue light with a dominant wavelength of 460.1 nm^38^ and illuminance of 3000 lux^35^ was applied. Besides, we installed a frequency regulator to stabilize the light at 40 Hz and also installed heat sinks on the box. Five weeks of uninterrupted light therapy protocol were performed for 3 h every day (8:00 a.m.–11:00 a.m.), and the rats could still get food and water freely during treatment.

### Statistical analysis

Data were expressed as mean ± SD and statistical analysis was accomplished using Stata/SE 12.0. Student *t* test was used in behavioral tests before light therapy, PET imaging analysis at baseline, and WB results. ANOVA with Bonferroni correction was used in behavioral tests and PET imaging analysis during and after light therapy. Pearson correlation coefficient was used to assess the correlation between SUV, BP_ND_-BLO, and BP_ND_-CBL. Significance levels were set at *p* < 0.05. Data were plotted using Origin 9.0.

## Results

### Depressive-like behavior was induced by CUMS and ameliorated by light therapy

**Fig. 1:**
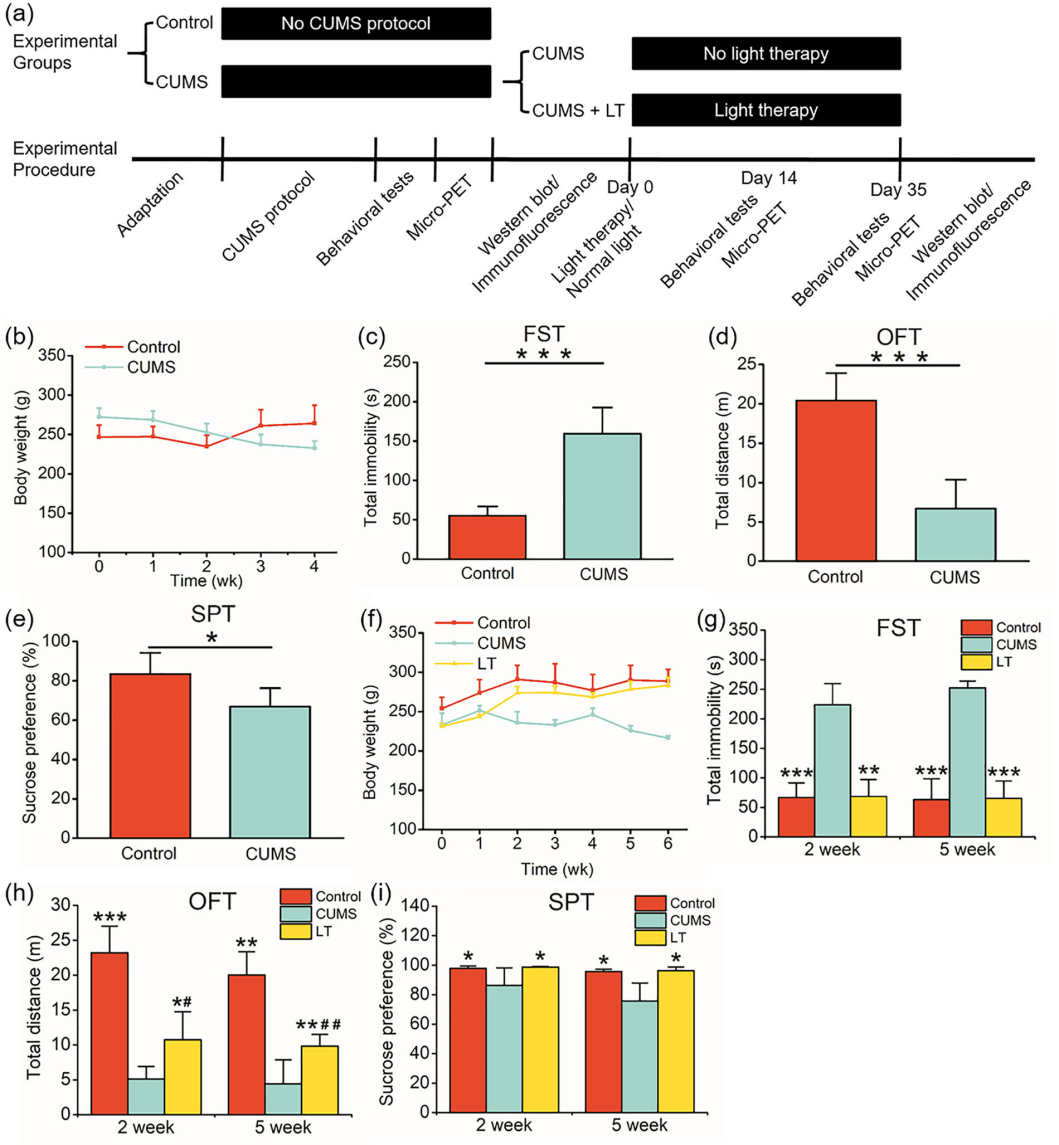
The results of behavioral tests. **a** The experimental design diagram. **b** The changes in body weight of rats during the modeling of CUMS. The results of the (**c**) FST, (**d**) OFT, and (**e**) SPT after CUMS modeling. **f** The changes in body weight of rats during light therapy. The results of the (**g**) FST, (**h**) OFT, and (**i**) SPT after 2 weeks and 5 weeks of light therapy. *p < 0.05; **p < 0.01; ***p < 0.001 compared with CUMS group; ^#^*p* < 0.05; ^##^*p* < 0.01 compared with control group. FST forced swimming test, OFT open field test, SPT sucrose preference test. *n* = 18/group for behavioral tests before light therapy (**b**–**e**). *n* = 6 (CUMS and LT) and *n* = 12 (control) for behavioral tests during and after light therapy (**f**–**i**).

During the modeling of CUMS, the body weight of CUMS rats showed a tendency to decrease. In contrast, the body weight of the rats in control group increased in fluctuation (Fig. 1b). After 4 weeks of modeling, CUMS rats showed a much longer total immobility time in the FST and a much shorter movement distance in the OFT compared to control group (Fig. 1c, d). Besides, the SPT results demonstrated that CUMS would lead to a slight but significant reduction of sucrose preference (Fig. 1e). During the treatment of light therapy, the body weight of the rats in LT group gradually increased and approached the control group, while the body weight of the untreated CUMS group decreased in fluctuation (Fig. 1f). As shown in Fig. 1g–i, after 2 weeks of light therapy, the total immobility time of LT group in the FST was almost the same as that of control group while the CUMS group showed no amelioration (Fig. 1g). Although the total movement distance of LT group in the OFT was still significantly different from control group, it has shown an increasing tendency and also a significant difference between LT and CUMS group (Fig. 1h). The results of the SPT showed close sucrose preference between LT and control group, and both of them were significantly higher than that of CUMS group (Fig. 1i). Besides, Fig. 1g–i showed that light therapy for 5 weeks or 2 weeks have similar effects on CUMS-induced depressive behavior.

### PET imaging analysis at baseline

**Fig. 2:**
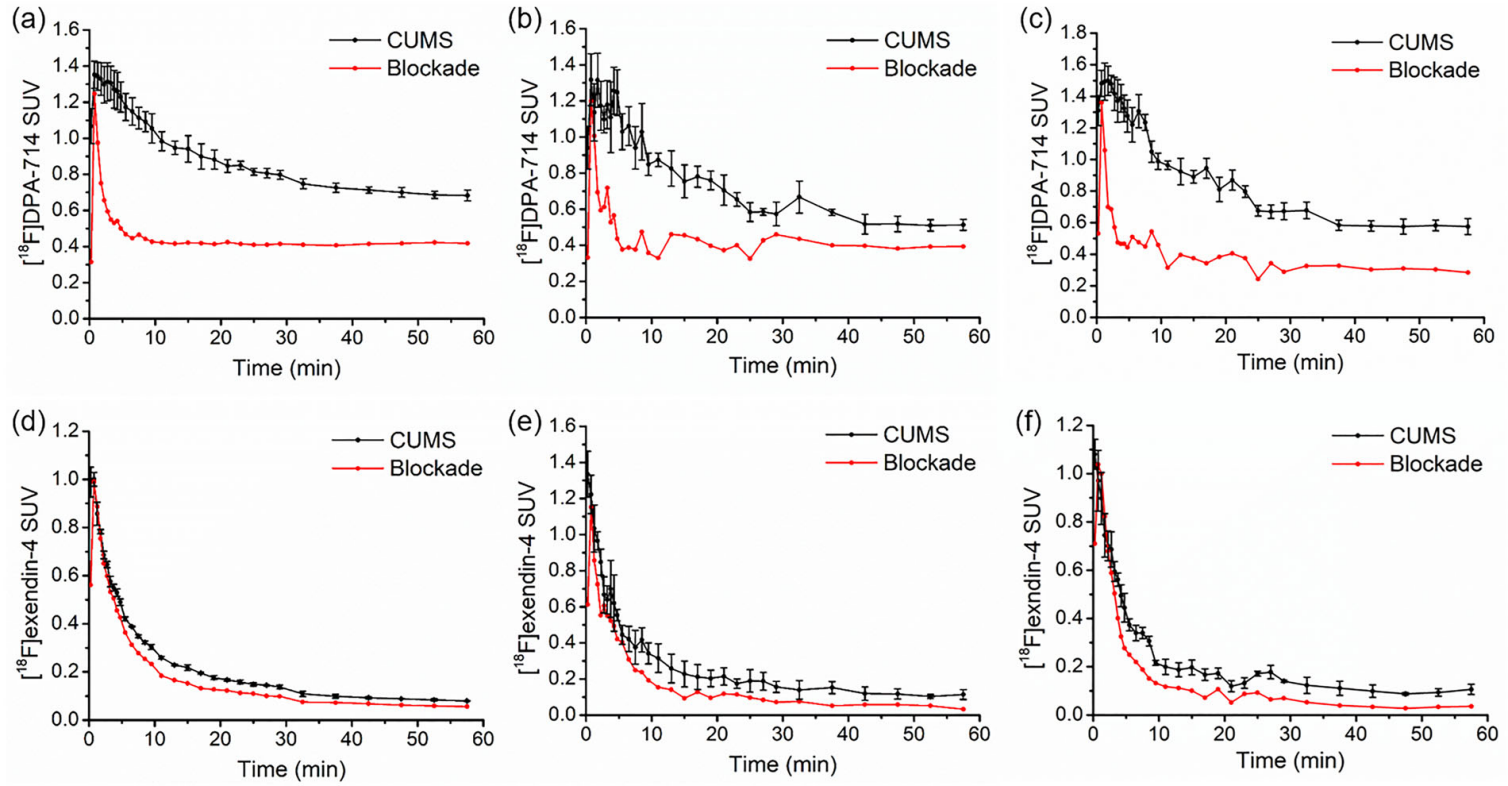
Dynamic PET analysis and blocking experiments of [^18^F]DPA-714 and [^18^F]exendin-4 in the brain of depressive rats. Time-activity curves of [^18^F]DPA-714 uptake in whole brain (**a**), posterior hippocampus (**b**), and medial prefrontal cortex (**c**). Time-activity curves of [^18^F]exendin-4 uptake in whole brain (**d**), hypothalamus (**e**), and ventral tegmental area (**f**). *n* = 4 for each group of dynamic experiments and *n* = 1 for each group of blocking experiments.

Time-activity curves representing the radioactive uptake of [^18^F]DPA-714 and [^18^F]exendin-4 in the brain were shown in Fig. 2. After bolus injection, both these two tracers reached their highest accumulation rapidly and decreased thereafter. The reduction of [^18^F]exendin-4 uptake was faster than that of [^18^F]DPA-714. After preinjection of non-radiolabeled precursor, the brain uptake of the two radiotracers quickly fell after reaching the peak, and the decrease in the uptake of the two radiotracers in the brain of the unblocked groups was significantly slower than that of blocked groups.

The BP_ND_-CBL and BP_ND_-BLO results of [^18^F]DPA-714 and [^18^F]exendin-4 in 33 ROIs of CUMS rats calculated by SRTM2 were shown in Supplementary Table S1. Both BP_ND_-CBL and BP_ND_-BLO correlated well with SUV for [^18^F]DPA-714 and [^18^F]exendin-4, and good correlations could also be found between BP_ND_-CBL and BP_ND_-BLO (Supplementary Table S2). For [^18^F]DPA-714, images derived from BP_ND_-BLO showed more detailed radioactive uptake distribution compared to images derived from BP_ND_-CBL, which was consistent with quantified data. For [^18^F]exendin-4, images derived from BP_ND_-CBL and BP_ND_-BLO both showed low uptake profile in the brain, which was also consistent with quantified data, however, the limited visual image quality of [^18^F]exendin-4 indicated that the visualization capability for this tracer needs to be further improved (Supplementary Fig. S1).

### Multimodal assessment of CUMS-induced depressive rats

#### [^18^F]DPA-714 PET

**Fig. 3:**
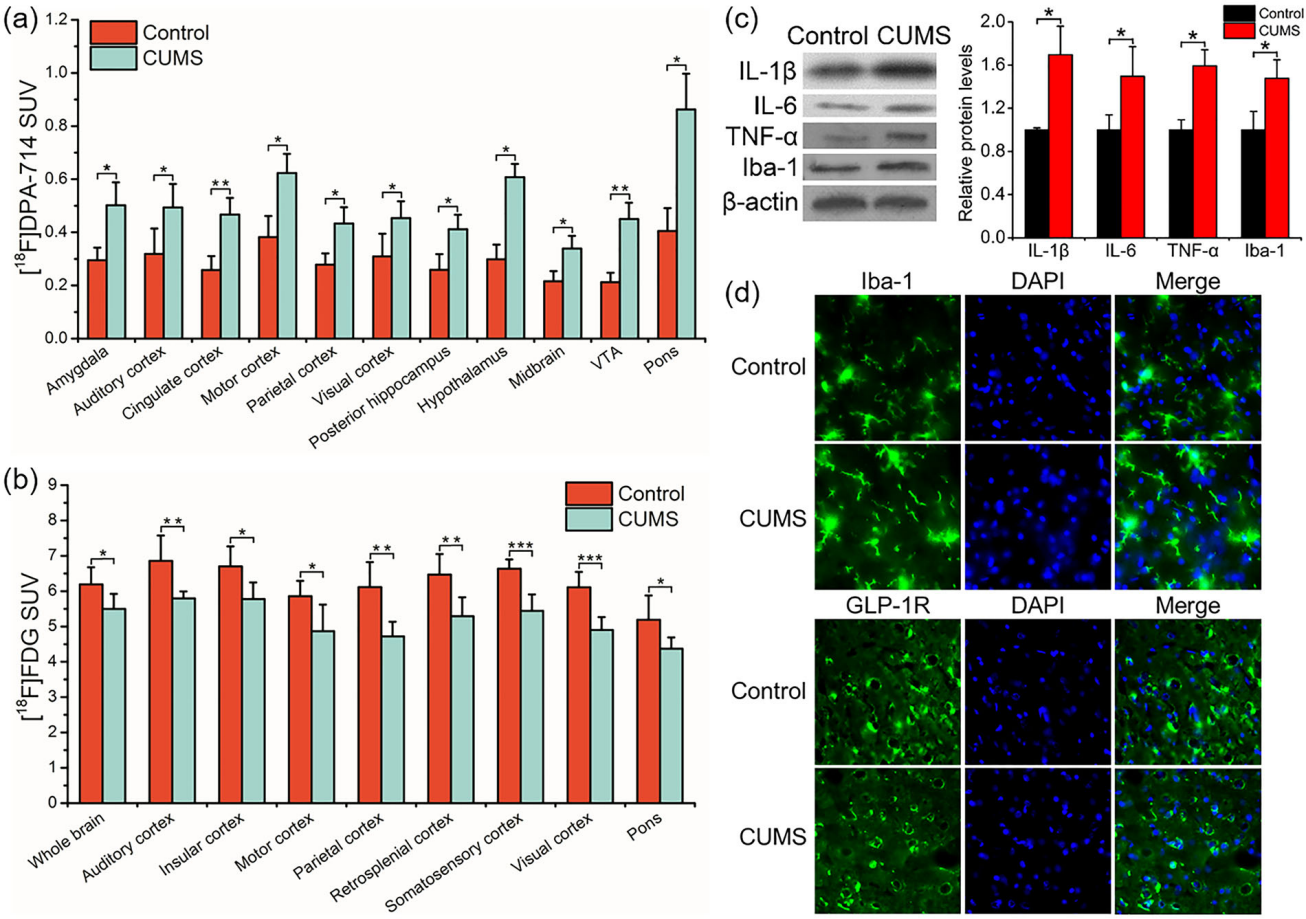
Multimodal assessment of CUMS-induced depressive rats. The [^18^F]DPA-714 uptake quantification in 11 ROIs (**a**) and the [^18^F]FDG uptake quantification in 9 ROIs (**b**) of the brain which showed significant differences between control group and CUMS group. **c** Western blot analysis of IL-1β, IL-6, TNF-α, and Iba-1 in hippocampus of normal rats and CUMS rats. **d** Immunofluorescence staining of hippocampal sections from normal rats and CUMS rats. *p < 0.05; **p < 0.01; ***p < 0.001. VTA ventral tegmental area. *n* = 12/group for PET imaging (**a**, **b**). *n* = 3 for western blot and immunofluorescence (**c**, **d**).

As shown in Fig. 3a, after the modeling period, CUMS rats had significant higher [^18^F]DPA-714 SUV compare to normal rats in 11 ROIs of the brain including amygdala, auditory cortex, cingulate cortex, motor cortex, parietal cortex, visual cortex, posterior hippocampus, hypothalamus, midbrain, ventral tegmental area, and pons (*p* < 0.05). Besides, although there were no significant differences, the [^18^F]DPA-714 uptake in most other brain regions of depressive rats was higher than that of normal rats, suggesting that depression would induce extensive microglial activation in the brain (Supplementary Table S3).

#### [^18^F]exendin-4 PET

As shown in Supplementary Table S3, there were no significant differences in [^18^F]exendin-4 uptake in most brain regions between CUMS and normal rats. However, the interesting higher accumulation of [^18^F]exendin-4 in auditory cortex and pituitary of CUMS rats than normal rats were found.

#### [^18^F]FDG PET

The widespread lower accumulation of [^18^F]FDG was observed in the brain of CUMS rats compared to normal rats (Supplementary Table S3), and significant differences existed in the whole brain, auditory cortex, insular cortex, motor cortex, parietal cortex, retrosplenial cortex, somatosensory cortex, visual cortex, and pons, indicating that CUMS-induced depression would lead to an extensive decline of glucose metabolism in the brain (Fig. 3b).

#### Western blot and immunofluorescence

Then, we evaluated the Iba-1 and inflammatory factors in the hippocampus using WB and IF. The results of WB showed elevated Iba-1, IL-6, IL-1β, and TNF-α levels in hippocampus of CUMS rats compared to normal rats (Fig. 3c). We also detected the higher expression of Iba-1, IL-6, and IL-1β in the hippocampus of CUMS rats using IF (Fig. 3d, Supplementary Fig. S2). These results were consistent with the quantification of PET imaging.

### Multimodal evaluation of light therapy for CUMS-induced depression

#### [^18^F]DPA-714 PET

After 2 weeks of light therapy, no significant differences in [^18^F]DPA-714 uptake existed among LT treated CUMS rats, untreated CUMS rats, and CUMS rats before LT (baseline) in all 33 ROIs (Supplementary Table S4). The same results were also found at 5 weeks after light therapy (Supplementary Table S5).

#### [^18^F]exendin-4 PET

**Fig. 4:**
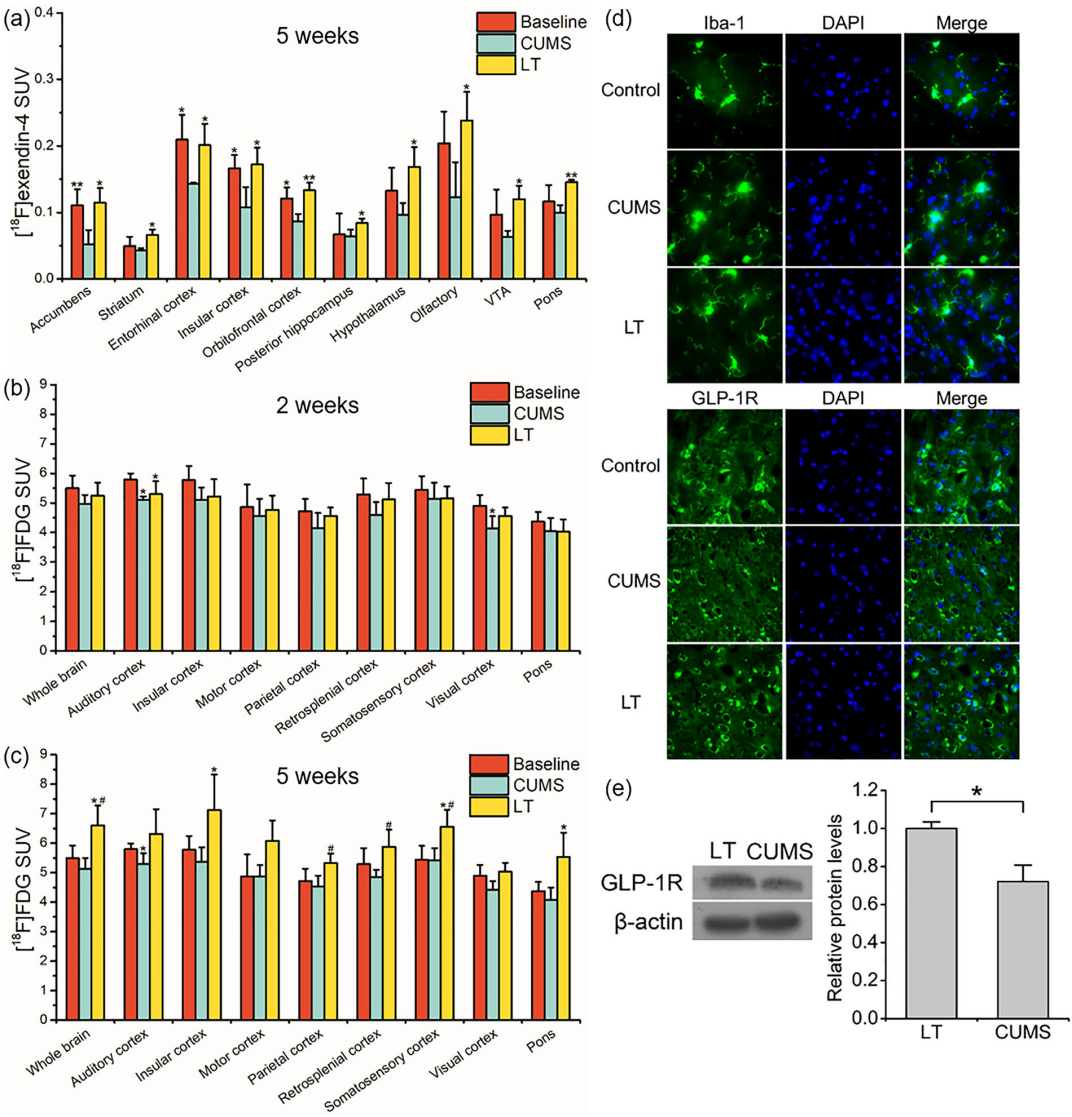
Multimodal evaluation of light therapy for CUMS-induced depression. **a** The [^18^F]exendin-4 quantification in ten ROIs that showed significant differences between CUMS and LT groups after 5 weeks of light therapy. *p < 0.05; **p < 0.01 compared to CUMS group. The [^18^F]FDG quantification after 2 weeks (**b**) and 5 weeks (**c**) of light therapy in nine ROIs which showed decreased glucose metabolism caused by CUMS at baseline. *p < 0.05 compared to baseline. ^#^*p* < 0.05 compared to CUMS group. **d** Immunofluorescence of hippocampal sections from normal rats, untreated CUMS rats, and LT treated CUMS rats. **e** Western blot analysis of GLP-1R in hippocampus of LT treated CUMS rats and untreated CUMS rats. *p < 0.05. VTA ventral tegmental area. *n* = 6 (CUMS and LT) and *n* = 12 (baseline) for PET imaging (**a**–**c**). *n* = 3 for western blot and immunofluorescence (**d**, **e**).

As shown in Supplementary Table S4, after 2 weeks of light therapy, there were no significant differences in [^18^F]exendin-4 uptake in all 33 ROIs of the brain between LT group and untreated CUMS group. Besides, neither of these two groups showed any significant differences compared to baseline. After 5 weeks of light therapy, significant lower uptake of [^18^F]exendin-4 was observed in untreated CUMS group compared to LT group in ten ROIs (*p* < 0.05). Besides, the [^18^F]exendin-4 uptake in five ROIs of untreated CUMS group was significantly lower than that of baseline. However, no significant differences in the [^18^F]exendin-4 uptake could be quantified in any ROI between LT group and baseline (Fig. 4a and Supplementary Table S5). These results suggested that light therapy prevented the decrease of GLP-1R expression in the brain of CUMS-induced depressive rats.

#### [^18^F]FDG PET

After 2 weeks of light therapy, although there were no significant differences, the [^18^F]FDG uptake in most ROIs of untreated CUMS group was lower than that of LT group. Besides, significantly lower uptake of [^18^F]FDG existed in ten ROIs of untreated CUMS group compared to baseline while significant different [^18^F]FDG uptake between LT group and baseline could only be observed in auditory cortex (Fig. 4b and Supplementary Table S4). After 5 weeks of light therapy, the [^18^F]FDG uptake in 24 ROIs of untreated CUMS group was significantly lower than that of LT group, besides, the [^18^F]FDG uptake in 20 ROIs of LT group was significantly higher than that of baseline. These results indicated that light therapy could reverse the extensive decreased glucose metabolism in the brain caused by CUMS (Fig. 4c and Supplementary Table S5).

#### Western blot and immunofluorescence

Since we observed a decreased [^18^F]exendin-4 uptake in the brain of untreated CUMS group compared to LT group, we validated the different expression of GLP-1R in the hippocampus between the two groups using WB and IF (Fig. 4d, e). Besides, IF staining of Iba-1 and inflammatory factors in hippocampal sections showed no obvious differences between CUMS group and LT group (Fig. 4d and Supplementary Fig. S3).

## Discussion

Recent studies confirmed that neuroinflammation plays an important role in the pathological process of depression. The evidence of microglial activation and neuroinflammation found in postmortem further supported this view^9,39^. PET as a functional imaging technique that has been widely used in neuropsychiatric disorders can visualize specific pathological processes noninvasively, dynamically, comprehensively, and has excellent quantitative capability^40^. In recent years, radiotracers targeting TSPO have been used in various neuropsychiatric disorders. Several clinical trials proved that these radiotracers have promising quantification ability for microglial activation in depression^13–15,17^. Our previous study proved for the first time that [^18^F]DPA-714 detected microglial activation in the hippocampus of depression rats^22^. In this present study, we further confirmed the neuroinflammatory changes induced by CUMS in multiple brain regions using [^18^F]DPA-714 PET. On the other hand, pathological experiments such as WB and IF are difficult to reflect the neuroinflammation in various regions of the brain at the same time. In this study, we subdivided the PET images of the whole brain into 32 regions and calculated the SUV for each ROI, thus obtained a relatively comprehensive whole brain inflammation profile.

Another primary strength of PET is its excellent quantification ability^41^. However, due to the small size of rodent brain and the wide expression of TSPO, the quantification of TSPO PET has always been a challenge^42^. There have been an increasing number of studies to explore the imaging data analysis methods for [^18^F]DPA-714^43–45^. Compartmental analysis is the gold standard for parameter estimation, however, this method is hard to perform in rodents since arterial blood sampling throughout the dynamic PET scan is difficult. Some radiotracers can use a reference region to estimate the binding parameters, but there is no suitable reference brain region for TSPO PET^46^. At present, the quantification for [^18^F]DPA-714 is to calculate SUV, SUV ratio (SUVr), or %ID/g^20^, and the parameter estimation can be performed by using cerebellum gray matter (GM) as a pseudo-reference region^43^. However, the quantitative imaging results in this study did not seem to support the use of GM as an ideal reference region. Therefore, we did not use SUVr to substitute SUV as the primary indicator. A better way to reflect the specific uptake of the radiotracer in the brain is to conduct parametric analysis to calculate total distribution volume (V_T_) or BP_ND_. In this study, we calculated BP_ND_ using GM as the pseudo-reference region (BP_ND_-CBL). Our blocking experiments validated the specific binding of [^18^F]DPA-714 as reported in previous studies^36^. We tried to use the data obtained in the blocking experiments as pseudo-reference regions to calculate BP_ND_ (BP_ND_-BLO). The results showed that there were significant correlations between BP_ND_-CBL, BP_ND_-BLO, and SUV (*p* < 0.001). Whether this method is reliable enough for parameter estimation need to be further validated using compartmental analysis. It should be noted that the use of blocking data to estimate the parameters is a calculation between two dynamic scans. Therefore, it is necessary to strictly control the same scan conditions and normalize the body weights and injection doses of the rats.

Recently, the antidepressant effects of GLP-1R agonists were proved^27,47^. A meta-analysis indicated that GLP-1 analogs resulted in a significant reduction of depression rating scales^28^. Besides, preclinical studies found that GLP-1 analogs and some other medications can modulate the GLP-1R expression in the brain^29^. However, there are few noninvasive methods available to assess the GLP-1R expression in the brain in vivo. Our previous study demonstrated that PET combined with radiolabeled exendin-4, a widely used GLP-1 analog, can be used for GLP-1R detection in vivo^32^. In this study, we evaluated the brain GLP-1R expression using ^18^F-labeled exendin-4 and the PET imaging results immediately after the CUMS modeling showed no significant differences in brain GLP-1R expression between CUMS group and control group. However, the quality of parametric images of [^18^F]exendin-4 PET was limited due to the low brain uptake and fast washout of the tracer showed in the dynamic results. The permeability of [^18^F]exendin-4 to cross the BBB is not ideal and the brain GLP-1R expression might be low, therefore, the quantification of imaging data would be essential. The blocking experiments in this study showed a slight but significant difference between blocking and unblocking groups which validated the specificity of this tracer in the brain. Then we did parameter estimation for [^18^F]exendin-4. Similar to [^18^F]DPA-714, the results indicated that cerebellum could not be an ideal true reference region. The definite expression pattern of brain GLP-1R expression is still unclear and its widespread expression might make it difficult to find a suitable reference region which is also similar to [^18^F]DPA-714.

Light therapy has been well accepted for the treatment of depression. In recent years, studies using [^11^C]harmine, [^11^C]DASB, and [^18^F]FDG PET proved that light therapy can modulate the levels of MAO-A, 5-HTT, and glucose metabolism in the brain of depression patients^6,7,48^. On the one hand, these studies provided useful information for understanding light therapy. On the other hand, they also proved that the noninvasive PET imaging has unique value and importance in monitoring pathological progress and guiding clinical medications of neuropsychiatric disorders. In this study, we used [^18^F]DPA-714 to evaluate the neuroinflammatory changes in the brain of CUMS-induced depression rats during light therapy. The results indicated that light therapy could not improve neuroinflammation caused by CUMS. The binding affinity and specificity of [^18^F]exendin-4 used in this study to GLP-1R have been validated previously in neuroendocrine tumors and pancreatic β-cell tracking^37,49^. Here, we used this tracer to evaluate the changes of brain GLP-1R expression during light therapy, and the results suggested that brain GLP-1R expression of untreated rats might continue to decrease within 5 weeks, while light therapy prevented the reduction. However, these results should be carefully considered since the brain uptake of [^18^F]exendin-4 is relatively low and no suitable quantification method has been established. We are currently doing more work to improve the efficiency of [^18^F]exendin-4 crossing the blood–brain barrier (BBB), so as to get more convincing quantification data and visualization performance.

This study had some limitations. For parameter estimation, we did not perform compartmental analysis because it was difficult to continuously gather blood samples during dynamic PET scans. However, SUV has also been validated as a promising indicator for PET imaging data quantification. The characteristics of [^18^F]exendin-4 for brain imaging need to be improved. We have compared several ^18^F-labeled exendin-4 and found that [^18^F]FBEM-Cys^39^-exendin-4 have higher BBB permeability than [^18^F]AlF-NOTA-MAL-Cys^39^-exendin-4^50^. However, the radiosynthesis of [^18^F]FBEM-Cys^39^-exendin-4 is much more complicated than [^18^F]AlF-NOTA-MAL-Cys^39^-exendin-4 and its yield is low^51^. Therefore, we think that [^18^F]FBEM-Cys^39^-exendin-4 might not be suitable for longitudinal dynamic PET scans of brain imaging. The high yield and simple synthesis of [^18^F]AlF-NOTA-MAL-Cys^39^-exendin-4 make it possible to perform multiple PET scans in a short period. Another point should be noted is that since the amount of imaging data is too large and the sensitivity of PET has a limit, the interpretation of the statistical significance needs to be cautious although we have conducted correction. In the Supplementary Tables, we list the SUV and their variances of all tracers in all regions at all time points, so as to make the display of the results more cautious and objective. Finally, a common limitation of all TSPO PET studies is that microglia have both pro-inflammatory and anti-inflammatory phenotypes, however, TSPO PET has limited ability to differentiate microglia between different phenotypes^52^.

In conclusion, we validated the widespread microglial activation and reduced glucose metabolism in the brain of depressive rats using PET imaging. Light therapy can ameliorate the depressive-like behavior, reverse the reduced glucose metabolism, and prevent the decline of GLP-1R expression. However, light therapy has no amelioration for the microglial activation in depression. Besides, the [^18^F]DPA-714 and [^18^F]exendin-4 PET showed quantitative potential in brain imaging, and these applications would be feasible and valuable for a better understanding of the pathophysiological process of depression and the clinical translational research in this field.

## Supplementary information

Supplementary Figure S1

Supplementary Figure S2

Supplementary Figure S3

Supplementary Table S1

Supplementary Table S2

Supplementary Table S3

Supplementary Table S4

Supplementary Table S5
