# Supplementary figures and images for: PET evaluation of light-induced modulation of microglial activation and GLP-1R expression in depressive rats
Source: Transl Psychiatry. 2021 Jan 6;11:26. doi: 10.1038/s41398-020-01155-z (PMC7791059; doi:10.1038/s41398-020-01155-z)

**
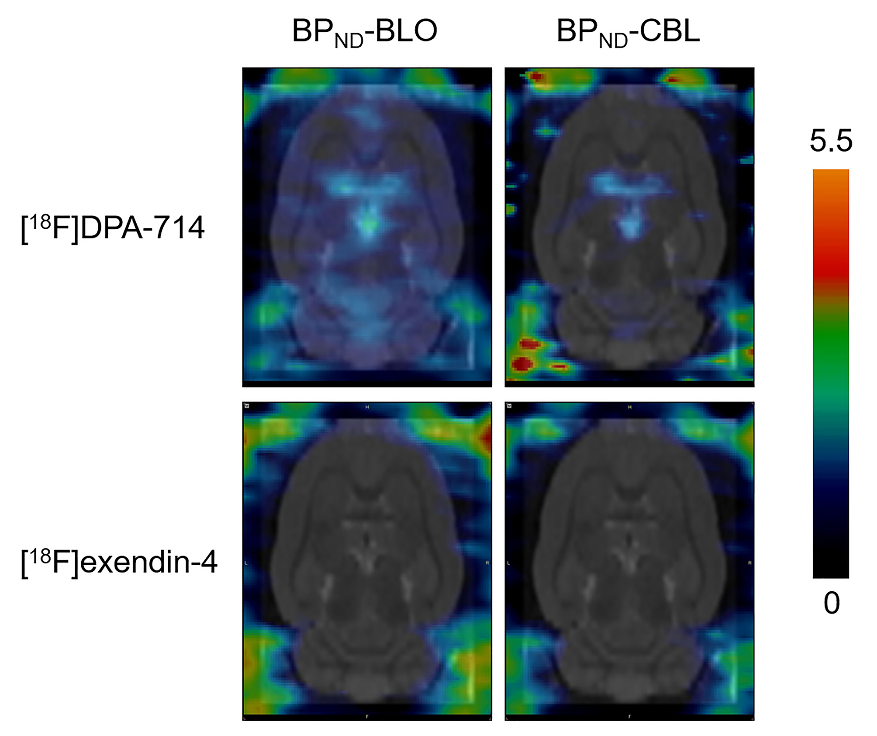
**

**Fig. S1** Parametric images of [18F]DPA-714 and [18F]exendin-4 in the brain of depressive rats.

Supplement: Supplementary file 1 — Supplementary Figure S1 [file 41398_2020_1155_MOESM1_ESM.docx]

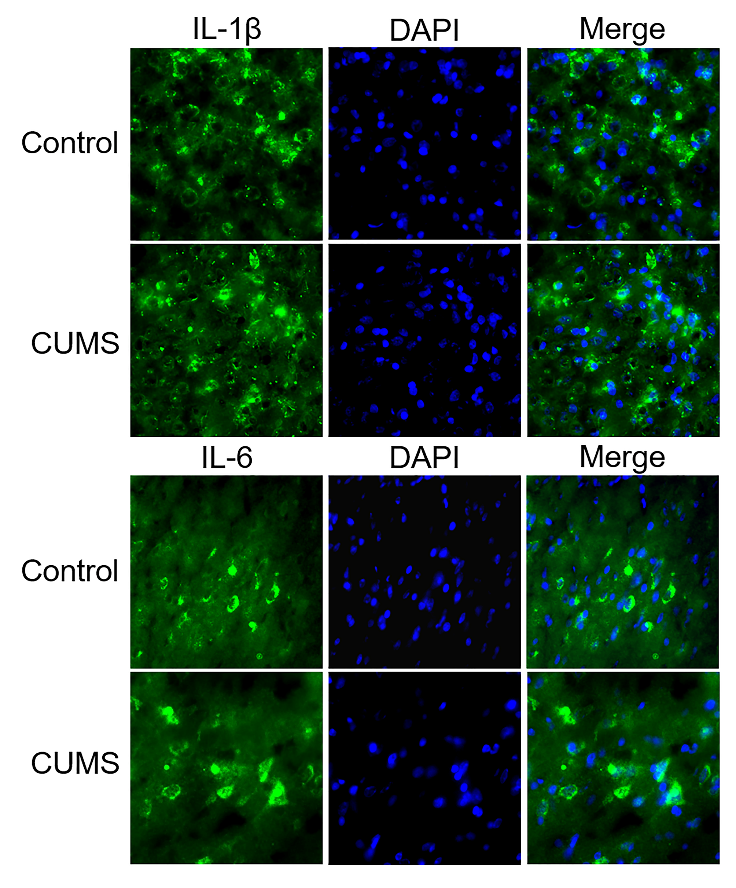


**Fig. S2** Immunofluorescence staining of hippocampal sections from normal rats and CUMS rats.

Supplement: Supplementary file 2 — Supplementary Figure S2 [file 41398_2020_1155_MOESM2_ESM.docx]
