# Supplementary material for: PET evaluation of light-induced modulation of microglial activation and GLP-1R expression in depressive rats
Source: Transl Psychiatry. 2021 Jan 6;11:26. doi: 10.1038/s41398-020-01155-z (PMC7791059; doi:10.1038/s41398-020-01155-z)
Supplement: Supplementary file 3 — Supplementary Figure S3 [file 41398_2020_1155_MOESM3_ESM.docx]

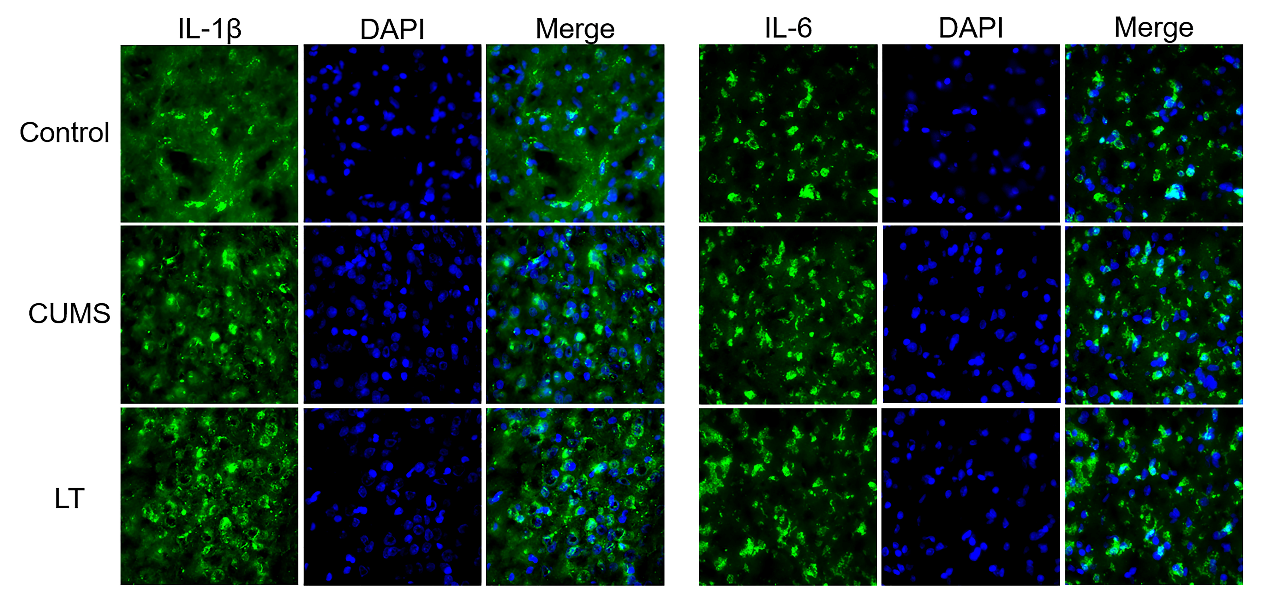


**Fig. S3** Immunofluorescence staining of hippocampal sections from normal rats, untreated CUMS rats, and LT treated CUMS rats after 5 weeks of light therapy.
