# Supplementary material for: PET evaluation of light-induced modulation of microglial activation and GLP-1R expression in depressive rats
Source: Transl Psychiatry. 2021 Jan 6;11:26. doi: 10.1038/s41398-020-01155-z (PMC7791059; doi:10.1038/s41398-020-01155-z)
Supplement: Supplementary file 4 — Supplementary Table S1 [file 41398_2020_1155_MOESM4_ESM.docx]

| **Table S1. BP_ND_ of [^18^F]DPA-714 and [^18^F]exendin-4 in the brain of depressive rats calculated using cerebellum and blocking as pseudo-reference region.** | | | | |
| --- | --- | --- | --- | --- |
|  | [^18^F]DPA-714 | | [^18^F]exendin-4 | |
|  | BP_ND_-CBL | BP_ND_-BLO | BP_ND_-CBL | BP_ND_-BLO |
| Whole brain | -0.03 (0.02) | 1.10 (0.11) | 0.10 (0.11) | 0.42 (0.08) |
| Accumbens | -0.51 (0.43) | 0.89 (0.11) | 0.21 (0.41) | 0.25 (0.10) |
| Amygdala | -0.11 (0.05) | 0.90 (0.18) | 0.31 (0.64) | 0.52 (0.11) |
| Striatum | 0.03 (0.32) | 1.01 (0.26) | -0.41 (0.05) | 0.29 (0.13) |
| Auditory cortex | -0.37 (0.03) | 0.67 (0.05) | 0.19 (0.47) | 0.31 (0.12) |
| Cingulate cortex | -0.40 (0.05) | 1.13 (0.34) | 0.11 (0.17) | 0.68 (0.32) |
| Entorhinal cortex | -0.05 (0.09) | 0.84 (0.09) | 0.67 (0.40) | 0.43 (0.24) |
| Frontal association cortex | 0.12 (0.30) | 0.61 (0.10) | 0.61 (0.26) | 0.01 (0.02) |
| Insular cortex | 0.00 (0.00) | 0.74 (0.12) | 0.32 (0.26) | 0.45 (0.17) |
| Medial prefrontal cortex | 0.14 (0.12) | 1.05 (0.25) | -0.10 (0.22) | 0.41 (0.14) |
| Motor cortex | -0.83 (0.30) | 1.00 (0.33) | 0.47 (0.25) | 0.55 (0.17) |
| Orbitofrontal cortex | -0.14 (0.22) | 0.78 (0.10) | 0.27 (0.23) | 0.16 (0.12) |
| Parietal cortex | 2.11 (3.94) | 0.95 (0.33) | 0.28 (0.31) | 0.55 (0.15) |
| Retrosplenial cortex | 1.62 (2.00) | 1.48 (0.49) | 0.48 (0.16) | 0.56 (0.22) |
| Somatosensory cortex | 0.45 (0.46) | 0.97 (0.18) | 0.12 (0.17) | 0.43 (0.11) |
| Visual cortex | 0.39 (0.50) | 1.02 (0.32) | 0.30 (0.23) | 0.53 (0.26) |
| Anterior dorsal hippocampus | -0.03 (0.12) | 0.42 (0.23) | -0.44 (0.05) | 0.23 (0.13) |
| Posterior hippocampus | 0.00 (0.32) | 1.05 (0.14) | -0.22 (0.35) | 0.23 (0.14) |
| Hypothalamus | 0.25 (0.32) | 1.60 (0.28) | 0.39 (0.60) | 0.70 (0.27) |
| Olfactory | -0.11 (0.23) | 0.67 (0.21) | 0.96 (0.61) | 0.46 (0.10) |
| Superior colliculus | 0.10 (0.31) | 1.20 (0.34) | -0.23 (0.09) | 0.42 (0.24) |
| Midbrain | -0.32 (0.11) | 1.67 (0.23) | -0.25 (0.07) | 0.40 (0.11) |
| Ventral tegmental area | -0.22 (0.68) | 1.46 (0.10) | 0.00 (0.27) | 0.46 (0.27) |
| Cerebellum-gray | 0.00 (0.00) | 1.12 (0.10) | 0.00 (0.00) | 0.31 (0.13) |
| Cerebellum-white | -0.77 (0.20) | 1.67 (0.11) | -0.26 (0.14) | 0.42 (0.19) |
| Inferior colliculus | -0.44 (0.13) | 1.10 (0.34) | -0.19 (0.29) | 0.20 (0.19) |
| Thalamus | -0.68 (0.28) | 1.42 (0.41) | -0.44 (0.14) | 0.21 (0.15) |
| Pituitary | 3.38 (0.34) | 3.37 (0.42) | 1.95 (0.52) | 1.13 (0.31) |
| Cerebellum-blood flow | 0.84 (0.14) | 2.18 (0.37) | -0.39 (0.13) | 0.25 (0.37) |
| Central canal | -0.82 (0.30) | 1.18 (0.43) | -0.39 (0.08) | 0.15 (0.09) |
| Pons | 0.39 (0.40) | 1.66 (0.32) | 0.30 (0.41) | 0.56 (0.20) |
| Septum | -0.48 (0.37) | 1.02 (0.54) | -0.51 (0.10) | 0.40 (0.15) |
| Medulla | 0.40 (0.47) | 1.72 (0.27) | -0.14 (0.26) | 0.31 (0.19) |

Data were presented as mean (SD).
