# Supplementary material for: PET evaluation of light-induced modulation of microglial activation and GLP-1R expression in depressive rats
Source: Transl Psychiatry. 2021 Jan 6;11:26. doi: 10.1038/s41398-020-01155-z (PMC7791059; doi:10.1038/s41398-020-01155-z)
Supplement: Supplementary file 5 — Supplementary Table S2 [file 41398_2020_1155_MOESM5_ESM.docx]

| **Table S2. Correlation coefficients of the three image parameters of [^18^F]DPA-714 and [^18^F]exendin-4 in the brain of depressive rats.** | | | | |
| --- | --- | --- | --- | --- |
|  | SUV | BP_ND_-CBL | BP_ND_-BLO | [^18^F]exendin-4 |
| SUV |  | 0.9638*** | 0.6164*** | SUV |
| BP_ND_-CBL | 0.6965*** |  | 0.6394*** | BP_ND_-CBL |
| BP_ND_-BLO | 0.8236*** | 0.5166** |  | BP_ND_-BLO |
| [^18^F]DPA-714 | SUV | BP_ND_-CBL | BP_ND_-BLO |  |

***P < 0.001.
