# Supplementary material for: PET evaluation of light-induced modulation of microglial activation and GLP-1R expression in depressive rats
Source: Transl Psychiatry. 2021 Jan 6;11:26. doi: 10.1038/s41398-020-01155-z (PMC7791059; doi:10.1038/s41398-020-01155-z)
Supplement: Supplementary file 6 — Supplementary Table S3 [file 41398_2020_1155_MOESM6_ESM.docx]

| **Table S3. Detailed data of the radioactive uptake in the ROIs of normal rats and CUMS-induced depressive rats.** | | | | | | | | | |
| --- | --- | --- | --- | --- | --- | --- | --- | --- | --- |
|  | [^18^F]DPA-714 SUV | | | [^18^F]exendin-4 SUV | | | [^18^F]FDG SUV | | |
|  | Control | CUMS | p-value | Control | CUMS | p-value | Control | CUMS | p-value |
| Whole brain | 0.346 (0.097) | 0.503 (0.105) | 0.055 | 0.109 (0.010) | 0.115 (0.015) | 0.517 | 6.190 (0.485) | 5.499 (0.422) | 0.032 |
| Accumbens | 0.220 (0.054) | 0.287 (0.085) | 0.218 | 0.112 (0.016) | 0.111 (0.014) | 0.930 | 7.061 (0.818) | 6.590 (0.732) | 0.339 |
| Amygdala | 0.296 (0.047) | 0.502 (0.086) | 0.040 | 0.116 (0.028) | 0.129 (0.021) | 0.064 | 5.341 (0.721) | 4.931 (0.555) | 0.313 |
| Striatum | 0.223 (0.057) | 0.273 (0.021) | 0.110 | 0.044 (0.002) | 0.050 (0.004) | 0.418 | 7.596 (0.726) | 6.852 (0.665) | 0.110 |
| Auditory cortex | 0.319 (0.096) | 0.494 (0.088) | 0.024 | 0.112 (0.016) | 0.143 (0.011) | 0.005 | 6.859 (0.716) | 5.798 (0.195) | 0.007 |
| Cingulate cortex | 0.258 (0.053) | 0.467 (0.063) | 0.003 | 0.097 (0.012) | 0.101 (0.012) | 0.353 | 7.476 (0.394) | 6.820 (0.923) | 0.175 |
| Entorhinal cortex | 0.395 (0.041) | 0.617 (0.170) | 0.075 | 0.177 (0.010) | 0.209 (0.017) | 0.134 | 5.691 (0.639) | 5.158 (0.402) | 0.125 |
| Frontal association cortex | 0.573 (0.137) | 0.673 (0.109) | 0.679 | 0.335 (0.068) | 0.177 (0.069) | 0.183 | 5.356 (1.378) | 3.796 (1.244) | 0.080 |
| Insular cortex | 0.323 (0.126) | 0.468 (0.116) | 0.116 | 0.160 (0.020) | 0.166 (0.020) | 0.627 | 6.702 (0.563) | 5.778 (0.469) | 0.015 |
| Medial prefrontal cortex | 0.262 (0.110) | 0.351 (0.140) | 0.337 | 0.114 (0.021) | 0.098 (0.020) | 0.250 | 8.002 (0.510) | 7.766 (0.706) | 0.549 |
| Motor cortex | 0.382 (0.080) | 0.623 (0.072) | 0.037 | 0.164 (0.034) | 0.146 (0.026) | 0.376 | 5.854 (0.435) | 4.868 (0.751) | 0.030 |
| Orbitofrontal cortex | 0.368 (0.108) | 0.429 (0.154) | 0.528 | 0.145 (0.024) | 0.121 (0.017) | 0.093 | 7.174 (0.491) | 6.382 (0.672) | 0.057 |
| Parietal cortex | 0.278 (0.042) | 0.433 (0.061) | 0.021 | 0.097 (0.012) | 0.108 (0.012) | 0.534 | 6.112 (0.708) | 4.721 (0.412) | 0.003 |
| Retrosplenial cortex | 0.375 (0.129) | 0.515 (0.099) | 0.107 | 0.126 (0.022) | 0.150 (0.020) | 0.111 | 6.469 (0.580) | 5.292 (0.535) | 0.007 |
| Somatosensory cortex | 0.305 (0.081) | 0.440 (0.121) | 0.097 | 0.102 (0.022) | 0.113 (0.023) | 0.476 | 6.635 (0.260) | 5.443 (0.462) | 0.001 |
| Visual cortex | 0.309 (0.086) | 0.453 (0.064) | 0.023 | 0.119 (0.014) | 0.125 (0.026) | 0.680 | 6.111 (0.433) | 4.901 (0.365) | 0.001 |
| Anterior dorsal hippocampus | 0.283 (0.070) | 0.360 (0.085) | 0.190 | 0.066 (0.003) | 0.045 (0.006) | 0.068 | 6.153 (0.767) | 6.043 (0.494) | 0.779 |
| Posterior hippocampus | 0.259 (0.060) | 0.412 (0.054) | 0.047 | 0.071 (0.015) | 0.067 (0.011) | 0.857 | 5.506 (0.450) | 5.198 (0.471) | 0.299 |
| Hypothalamus | 0.299 (0.055) | 0.608 (0.050) | 0.012 | 0.091 (0.016) | 0.132 (0.015) | 0.059 | 5.528 (0.811) | 5.112 (0.466) | 0.313 |
| Olfactory | 0.464 (0.087) | 0.575 (0.078) | 0.312 | 0.175 (0.017) | 0.204 (0.018) | 0.286 | 6.235 (0.639) | 5.832 (0.443) | 0.248 |
| Superior colliculus | 0.300 (0.053) | 0.349 (0.051) | 0.495 | 0.085 (0.017) | 0.064 (0.009) | 0.237 | 7.181 (0.851) | 6.523 (0.504) | 0.145 |
| Midbrain | 0.216 (0.039) | 0.339 (0.048) | 0.036 | 0.062 (0.018) | 0.055 (0.005) | 0.574 | 6.775 (0.815) | 6.175 (0.574) | 0.185 |
| Ventral tegmental area | 0.212 (0.036) | 0.450 (0.061) | 0.002 | 0.086 (0.016) | 0.097 (0.018) | 0.369 | 6.191 (0.652) | 5.441 (0.625) | 0.084 |
| Cerebellum-gray | 0.414 (0.064) | 0.492 (0.058) | 0.250 | 0.120 (0.020) | 0.098 (0.018) | 0.098 | 4.813 (0.396) | 4.642 (0.331) | 0.454 |
| Cerebellum-white | 0.480 (0.070) | 0.623 (0.100) | 0.115 | 0.066 (0.012) | 0.062 (0.014) | 0.622 | 5.866 (0.565) | 5.590 (0.463) | 0.396 |
| Inferior colliculus | 0.294 (0.054) | 0.310 (0.048) | 0.773 | 0.096 (0.020) | 0.099 (0.015) | 0.893 | 7.963 (1.297) | 6.849 (0.536) | 0.086 |
| Thalamus | 0.276 (0.049) | 0.335 (0.057) | 0.362 | 0.039 (0.007) | 0.044 (0.011) | 0.423 | 6.941 (0.746) | 6.502 (0.537) | 0.285 |
| Pituitary | 1.360 (0.320) | 2.062 (0.331) | 0.068 | 0.216 (0.024) | 0.404 (0.047) | 0.020 | 3.443 (0.722) | 2.706 (0.425) | 0.064 |
| Cerebellum-blood flow | 0.865 (0.131) | 0.819 (0.118) | 0.818 | 0.037 (0.011) | 0.045 (0.015) | 0.079 | 7.268 (0.864) | 7.132 (0.559) | 0.759 |
| Central canal | 0.171 (0.075) | 0.267 (0.062) | 0.188 | 0.045 (0.013) | 0.044 (0.007) | 0.967 | 6.568 (0.608) | 5.958 (0.435) | 0.084 |
| Pons | 0.405 (0.086) | 0.862 (0.135) | 0.033 | 0.110 (0.029) | 0.116 (0.025) | 0.749 | 5.189 (0.686) | 4.371 (0.319) | 0.028 |
| Septum | 0.379 (0.075) | 0.358 (0.060) | 0.793 | 0.046 (0.007) | 0.050 (0.012) | 0.771 | 6.079 (0.658) | 5.403 (0.607) | 0.110 |
| Medulla | 0.479 (0.080) | 0.776 (0.181) | 0.198 | 0.091 (0.012) | 0.091 (0.020) | 0.998 | 5.831 (0.659) | 5.031 (0.573) | 0.059 |

Data were presented as mean (SD) and p-value were calculated by two-sample t test.
