# Supplementary material for: PET evaluation of light-induced modulation of microglial activation and GLP-1R expression in depressive rats
Source: Transl Psychiatry. 2021 Jan 6;11:26. doi: 10.1038/s41398-020-01155-z (PMC7791059; doi:10.1038/s41398-020-01155-z)
Supplement: Supplementary file 7 — Supplementary Table S4 [file 41398_2020_1155_MOESM7_ESM.docx]

| **Table S4. Detailed data of the radioactive uptake in the ROIs of LT treated rats and untreated rats after 2 weeks of light therapy.** | | | | | | | | | |
| --- | --- | --- | --- | --- | --- | --- | --- | --- | --- |
|  | [^18^F]DPA-714 SUV | | | [^18^F]exendin-4 SUV | | | [^18^F]FDG SUV | | |
|  | LT | CUMS | p-value | LT | CUMS | p-value | LT | CUMS | p-value |
| Whole brain | 0.550 (0.164) | 0.536 (0.094) | 0.901 | 0.115 (0.010) | 0.102 (0.015) | 0.275 | 5.249 (0.436) | 4.966 (0.298) | 0.406 |
| Accumbens | 0.316 (0.035) | 0.288 (0.069) | 0.571 | 0.105 (0.022) | 0.107 (0.026) | 0.096 | 5.931 (0.702) | 5.541* (0.071) | 0.392 |
| Amygdala | 0.512 (0.161) | 0.455 (0.098) | 0.307 | 0.136 (0.006) | 0.123 (0.028) | 0.357 | 4.564 (0.483) | 4.122* (0.184) | 0.213 |
| Striatum | 0.315 (0.074) | 0.334 (0.076) | 0.780 | 0.045 (0.006) | 0.033 (0.006) | 0.069 | 6.312 (0.615) | 5.911 (0.354) | 0.383 |
| Auditory cortex | 0.592 (0.147) | 0.489 (0.029) | 0.300 | 0.140 (0.024) | 0.128 (0.027) | 0.595 | 5.298* (0.436) | 5.107* (0.115) | 0.505 |
| Cingulate cortex | 0.452 (0.191) | 0.524 (0.184) | 0.862 | 0.114 (0.005) | 0.096 (0.046) | 0.233 | 6.416 (0.538) | 5.983 (0.518) | 0.372 |
| Entorhinal cortex | 0.666 (0.231) | 0.603 (0.126) | 0.700 | 0.191 (0.013) | 0.182 (0.030) | 0.659 | 4.671 (0.450) | 4.392* (0.101) | 0.355 |
| Frontal association cortex | 0.635 (0.162) | 0.637 (0.110) | 0.748 | 0.194 (0.087) | 0.179 (0.039) | 0.085 | 4.443 (0.276) | 4.684 (0.654) | 0.589 |
| Insular cortex | 0.538 (0.142) | 0.458 (0.049) | 0.407 | 0.142 (0.029) | 0.125 (0.038) | 0.571 | 5.215 (0.585) | 5.111 (0.410) | 0.813 |
| Medial prefrontal cortex | 0.415 (0.177) | 0.382 (0.117) | 0.797 | 0.080 (0.035) | 0.039 (0.015) | 0.132 | 6.833 (0.654) | 6.520* (0.315) | 0.497 |
| Motor cortex | 0.708 (0.372) | 0.766 (0.213) | 0.828 | 0.207 (0.039) | 0.118 (0.030) | 0.056 | 4.759 (0.484) | 4.566 (0.573) | 0.678 |
| Orbitofrontal cortex | 0.518 (0.197) | 0.510 (0.073) | 0.950 | 0.131 (0.002) | 0.107 (0.017) | 0.074 | 5.937 (0.584) | 5.848 (0.415) | 0.840 |
| Parietal cortex | 0.465 (0.176) | 0.472 (0.148) | 0.960 | 0.112 (0.022) | 0.100 (0.044) | 0.538 | 4.561 (0.282) | 4.151 (0.510) | 0.290 |
| Retrosplenial cortex | 0.526 (0.174) | 0.582 (0.290) | 0.790 | 0.157 (0.011) | 0.147 (0.012) | 0.378 | 5.119 (0.553) | 4.596 (0.428) | 0.265 |
| Somatosensory cortex | 0.541 (0.236) | 0.533 (0.156) | 0.964 | 0.132 (0.019) | 0.098 (0.030) | 0.182 | 5.153 (0.408) | 5.138 (0.543) | 0.972 |
| Visual cortex | 0.539 (0.135) | 0.572 (0.175) | 0.810 | 0.136 (0.062) | 0.133 (0.028) | 0.593 | 4.563 (0.283) | 4.131* (0.421) | 0.215 |
| Anterior dorsal hippocampus | 0.360 (0.111) | 0.345 (0.023) | 0.822 | 0.053 (0.008) | 0.044 (0.005) | 0.162 | 5.719 (0.661) | 5.256* (0.120) | 0.298 |
| Posterior hippocampus | 0.454 (0.159) | 0.395 (0.084) | 0.601 | 0.063 (0.016) | 0.060 (0.031) | 0.839 | 4.951 (0.513) | 4.498* (0.149) | 0.216 |
| Hypothalamus | 0.571 (0.117) | 0.588 (0.059) | 0.335 | 0.159 (0.014) | 0.123 (0.016) | 0.052 | 4.743 (0.509) | 4.403* (0.186) | 0.339 |
| Olfactory | 0.606 (0.054) | 0.591 (0.137) | 0.864 | 0.145 (0.018) | 0.127 (0.030) | 0.202 | 5.449 (0.673) | 5.098* (0.147) | 0.426 |
| Superior colliculus | 0.389 (0.062) | 0.391 (0.122) | 0.981 | 0.071 (0.018) | 0.039 (0.010) | 0.054 | 6.387 (0.469) | 5.892 (0.346) | 0.215 |
| Midbrain | 0.325 (0.053) | 0.333 (0.062) | 0.870 | 0.064 (0.010) | 0.050 (0.015) | 0.257 | 5.981 (0.481) | 5.514 (0.271) | 0.217 |
| Ventral tegmental area | 0.475 (0.085) | 0.417 (0.045) | 0.354 | 0.075 (0.030) | 0.092 (0.039) | 0.597 | 5.113 (0.442) | 4.623 (0.212) | 0.159 |
| Cerebellum-gray | 0.564 (0.133) | 0.556 (0.062) | 0.933 | 0.096 (0.016) | 0.085 (0.018) | 0.467 | 4.972 (0.381) | 4.657 (0.362) | 0.357 |
| Cerebellum-white | 0.591 (0.159) | 0.662 (0.133) | 0.584 | 0.065 (0.008) | 0.050 (0.002) | 0.054 | 5.699 (0.484) | 5.348 (0.374) | 0.377 |
| Inferior colliculus | 0.379 (0.074) | 0.359 (0.082) | 0.769 | 0.077 (0.021) | 0.086 (0.061) | 0.816 | 6.513 (0.617) | 6.114 (0.629) | 0.476 |
| Thalamus | 0.432 (0.106) | 0.409 (0.128) | 0.825 | 0.042 (0.009) | 0.046 (0.007) | 0.571 | 6.559 (0.589) | 6.303 (0.252) | 0.526 |
| Pituitary | 2.085 (0.734) | 2.383 (0.451) | 0.581 | 0.317 (0.092) | 0.365 (0.132) | 0.636 | 2.291 (0.209) | 2.298 (0.141) | 0.962 |
| Cerebellum-blood flow | 1.026 (0.145) | 0.828 (0.324) | 0.387 | 0.063 (0.048) | 0.039 (0.040) | 0.110 | 7.023 (0.372) | 6.577 (0.074) | 0.111 |
| Central canal | 0.316 (0.110) | 0.293 (0.078) | 0.787 | 0.042 (0.026) | 0.037 (0.006) | 0.814 | 6.059 (0.408) | 5.605 (0.174) | 0.151 |
| Pons | 0.654 (0.251) | 0.655 (0.075) | 0.993 | 0.143 (0.010) | 0.119 (0.025) | 0.177 | 4.024 (0.414) | 4.042 (0.452) | 0.963 |
| Septum | 0.445 (0.195) | 0.555 (0.337) | 0.651 | 0.058 (0.018) | 0.055 (0.037) | 0.919 | 5.121 (0.440) | 4.478 (0.032) | 0.065 |
| Medulla | 0.701 (0.203) | 0.672 (0.102) | 0.840 | 0.083 (0.008) | 0.079 (0.013) | 0.634 | 4.777 (0.540) | 4.554 (0.565) | 0.647 |

Data were presented as mean (SD) and p-value were calculated by two-sample t test. *p < 0.05 compared with baseline.
