# Supplementary material for: PET evaluation of light-induced modulation of microglial activation and GLP-1R expression in depressive rats
Source: Transl Psychiatry. 2021 Jan 6;11:26. doi: 10.1038/s41398-020-01155-z (PMC7791059; doi:10.1038/s41398-020-01155-z)
Supplement: Supplementary file 8 — Supplementary Table S5 [file 41398_2020_1155_MOESM8_ESM.docx]

| **Table S5. Detailed data of the radioactive uptake in the ROIs of LT treated rats and untreated rats after 5 weeks of light therapy.** | | | | | | | | | |
| --- | --- | --- | --- | --- | --- | --- | --- | --- | --- |
|  | [^18^F]DPA-714 SUV | | | [^18^F]exendin-4 SUV | | | [^18^F]FDG SUV | | |
|  | LT | CUMS | p-value | LT | CUMS | p-value | LT | CUMS | p-value |
| Whole brain | 0.510 (0.098) | 0.538 (0.092) | 0.739 | 0.117 (0.015) | 0.100 (0.014) | 0.231 | 6.598* (0.673) | 5.131 (0.361) | 0.029 |
| Accumbens | 0.299 (0.055) | 0.297 (0.065) | 0.970 | 0.115 (0.022) | 0.052* (0.021) | 0.024 | 7.706 (0.868) | 5.554 (0.540) | 0.022 |
| Amygdala | 0.504 (0.026) | 0.437 (0.075) | 0.216 | 0.153 (0.031) | 0.110* (0.005) | 0.076 | 5.712 (0.679) | 4.147 (0.172) | 0.018 |
| Striatum | 0.317 (0.046) | 0.305 (0.041) | 0.828 | 0.066 (0.008) | 0.043 (0.003) | 0.011 | 7.962 (0.911) | 6.190 (0.504) | 0.042 |
| Auditory cortex | 0.632 (0.216) | 0.570 (0.107) | 0.678 | 0.167 (0.028) | 0.138 (0.015) | 0.189 | 6.309 (0.846) | 5.298* (0.359) | 0.129 |
| Cingulate cortex | 0.454 (0.022) | 0.463 (0.116) | 0.899 | 0.093 (0.007) | 0.107 (0.040) | 0.595 | 8.482* (1.026) | 6.376 (0.448) | 0.031 |
| Entorhinal cortex | 0.616 (0.146) | 0.707 (0.178) | 0.644 | 0.201 (0.032) | 0.143* (0.002) | 0.034 | 5.960 (0.938) | 4.409* (0.364) | 0.056 |
| Frontal association cortex | 0.572 (0.052) | 0.639 (0.117) | 0.630 | 0.143 (0.008) | 0.176 (0.035) | 0.186 | 6.053 (0.852) | 5.026 (0.629) | 0.168 |
| Insular cortex | 0.590 (0.081) | 0.560 (0.109) | 0.845 | 0.172 (0.025) | 0.108* (0.030) | 0.046 | 7.124* (1.200) | 5.361 (0.498) | 0.079 |
| Medial prefrontal cortex | 0.340 (0.048) | 0.420 (0.069) | 0.474 | 0.064 (0.024) | 0.053 (0.024) | 0.580 | 9.072* (0.920) | 6.685 (0.865) | 0.031 |
| Motor cortex | 0.547 (0.094) | 0.577 (0.113) | 0.740 | 0.148 (0.008) | 0.149 (0.053) | 0.257 | 6.081 (0.690) | 4.864 (0.389) | 0.056 |
| Orbitofrontal cortex | 0.444 (0.071) | 0.499 (0.030) | 0.279 | 0.133 (0.012) | 0.087* (0.011) | 0.007 | 7.944* (0.937) | 6.075 (0.538) | 0.040 |
| Parietal cortex | 0.517 (0.102) | 0.524 (0.070) | 0.931 | 0.107 (0.029) | 0.105 (0.030) | 0.482 | 5.326 (0.322) | 4.520 (0.374) | 0.047 |
| Retrosplenial cortex | 0.560 (0.115) | 0.638 (0.101) | 0.577 | 0.134 (0.015) | 0.151 (0.061) | 0.373 | 5.878 (0.575) | 4.845 (0.251) | 0.046 |
| Somatosensory cortex | 0.504 (0.075) | 0.528 (0.081) | 0.835 | 0.110 (0.015) | 0.109 (0.041) | 0.967 | 6.556* (0.574) | 5.410 (0.423) | 0.049 |
| Visual cortex | 0.539 (0.135) | 0.520 (0.085) | 0.847 | 0.121 (0.009) | 0.120 (0.044) | 0.699 | 5.036 (0.296) | 4.425 (0.279) | 0.060 |
| Anterior dorsal hippocampus | 0.334 (0.043) | 0.352 (0.054) | 0.767 | 0.051 (0.004) | 0.050 (0.013) | 0.917 | 7.655* (0.937) | 5.323 (0.402) | 0.017 |
| Posterior hippocampus | 0.403 (0.070) | 0.410 (0.070) | 0.913 | 0.084 (0.007) | 0.064 (0.010) | 0.047 | 6.397* (0.879) | 4.702 (0.219) | 0.032 |
| Hypothalamus | 0.582 (0.131) | 0.514 (0.141) | 0.574 | 0.168 (0.030) | 0.096 (0.018) | 0.023 | 6.352* (0.629) | 4.447 (0.314) | 0.009 |
| Olfactory | 0.625 (0.076) | 0.604 (0.098) | 0.877 | 0.238 (0.043) | 0.123 (0.052) | 0.043 | 7.117* (0.739) | 4.925* (0.246) | 0.008 |
| Superior colliculus | 0.348 (0.028) | 0.362 (0.007) | 0.437 | 0.063 (0.016) | 0.051 (0.015) | 0.418 | 7.912* (0.864) | 6.037 (0.438) | 0.028 |
| Midbrain | 0.319 (0.044) | 0.318 (0.051) | 0.986 | 0.068 (0.009) | 0.049 (0.010) | 0.064 | 7.631* (0.965) | 5.800 (0.409) | 0.039 |
| Ventral tegmental area | 0.429 (0.068) | 0.326 (0.128) | 0.284 | 0.120 (0.020) | 0.064 (0.009) | 0.011 | 6.403* (0.661) | 4.704 (0.458) | 0.022 |
| Cerebellum-gray | 0.543 (0.098) | 0.548 (0.053) | 0.939 | 0.098 (0.021) | 0.088 (0.013) | 0.513 | 5.711* (0.151) | 4.717 (0.418) | 0.018 |
| Cerebellum-white | 0.541 (0.079) | 0.580 (0.033) | 0.690 | 0.070 (0.010) | 0.057 (0.021) | 0.375 | 6.785* (0.513) | 5.478 (0.380) | 0.024 |
| Inferior colliculus | 0.391 (0.084) | 0.413 (0.027) | 0.820 | 0.074 (0.039) | 0.079 (0.006) | 0.815 | 7.783 (1.173) | 6.336 (0.277) | 0.106 |
| Thalamus | 0.362 (0.035) | 0.391 (0.068) | 0.541 | 0.042 (0.010) | 0.044 (0.006) | 0.783 | 8.414* (1.014) | 6.474 (0.547) | 0.043 |
| Pituitary | 1.843 (0.215) | 2.403 (0.254) | 0.198 | 0.338 (0.028) | 0.342 (0.056) | 0.058 | 3.410 (0.407) | 2.107 (0.053) | 0.005 |
| Cerebellum-blood flow | 0.927 (0.139) | 0.710 (0.259) | 0.272 | 0.063 (0.049) | 0.032 (0.026) | 0.387 | 8.843* (0.573) | 6.623 (0.539) | 0.008 |
| Central canal | 0.308 (0.100) | 0.300 (0.100) | 0.921 | 0.036 (0.035) | 0.032 (0.010) | 0.864 | 7.666* (0.843) | 5.532 (0.521) | 0.020 |
| Pons | 0.535 (0.047) | 0.710 (0.159) | 0.313 | 0.146 (0.004) | 0.100 (0.011) | 0.003 | 5.532* (0.817) | 4.082 (0.415) | 0.052 |
| Septum | 0.371 (0.099) | 0.368 (0.101) | 0.965 | 0.054 (0.017) | 0.034 (0.013) | 0.188 | 6.651* (0.832) | 4.865 (0.382) | 0.028 |
| Medulla | 0.742 (0.138) | 0.866 (0.314) | 0.506 | 0.090 (0.009) | 0.096 (0.017) | 0.621 | 6.311* (0.903) | 4.762 (0.396) | 0.053 |

Data were presented as mean (SD) and p-value were calculated by two-sample t test. *p < 0.05 compared with baseline.
